# The Value of SPECT/CT in Monitoring Prefabricated Tissue-Engineered Bone and Orthotopic rhBMP-2 Implants for Mandibular Reconstruction

**DOI:** 10.1371/journal.pone.0137167

**Published:** 2015-09-04

**Authors:** Miao Zhou, Xin Peng, Chi Mao, Jia-he Tian, Shu-wen Zhang, Fang Xu, Jing-jing Tu, Sheng Liu, Min Hu, Guang-yan Yu

**Affiliations:** 1 Department of Oral and Maxillofacial Surgery, Peking University School and Hospital of Stomatology, Beijing, P.R. China; 2 Key Laboratory of Oral Medicine, Guangzhou Institute of Oral Disease, Stomatology Hospital of Guangzhou Medical University, Guangzhou, P.R. China; 3 Department of Nuclear Medicine, Chinese PLA General Hospital, Beijing, P.R. China; 4 College of Pharmaceutical Science, Zhejiang University of Technology, Hangzhou, P.R. China; 5 Department of Nuclear Medicine, Sun Yat-sen Memorial Hospital, Sun Yat-sen University, Guangzhou, P.R. China; 6 Department of Oral and Maxillofacial Surgery, Chinese PLA General Hospital, Beijing, P.R. China; Medical University of South Carolina, UNITED STATES

## Abstract

Bone tissue engineering shows good prospects for mandibular reconstruction. In recent studies, prefabricated tissue-engineered bone (PTEB) by recombinant human bone morphogenetic proteins (rhBMPs) applied in vivo has found to be an effective alternative for autologous bone grafts. However, the optimal time to transfer PTEB for mandibular reconstruction is still not elucidated. Thus, here in an animal experiment of rhesus monkey, the suitable transferring time for PTEB to reconstruct mandibular defects was evaluated by ^99m^Tc-MDP SPECT/CT, and its value in monitoring orthotopic rhBMP-2 implants for mandibular reconstruction was also evaluated. The result of SPECT/CT showed higher ^99m^Tc-MDP uptake, indicating osteoinductivity, in rhBMP-2 incorporated demineralized freeze-dried bone allograft (DFDBA) and coralline hydroxyapatite (CHA) implants than those without BMP stimulation. ^99m^Tc-MDP uptake of rhBMP-2 implant peaked at 8 weeks following implantation while CT showed the density of these implants increased after 13 weeks’ prefabrication. Histology confirmed that mandibular defects were repaired successfully with PTEB or orthotopically rhBMP-2 incorporated CHA implants, in accordance with SPECT/CT findings. Collectively, data shows ^99m^Tc-MDP SPECT/CT is a sensitive and noninvasive tool to monitor osteoinductivity and bone regeneration of PTEB and orthotopic implants. The PTEB achieved peak osteoinductivity and bone density at 8 to 13 weeks following ectopic implantation, which would serve as a recommendable time frame for its transfer to mandibular reconstruction.

## Introduction

With the increasing number of patients suffering from malignant head and neck tumors with a need for surgical resection of the mandible, the demand for bone graft procedures is rising. The repair of mandibular defects entails the application of autograft, allograft and xenograft. Though autograft is still considered the best method of repair, the clinical application of autologous bone grafts is limited by the availability of bone tissue. New methods for bone regeneration are, therefore in need [1].

Bone morphogenetic proteins (BMPs) have been used in non-union, fracture of the bone etc [2]. Prefabricated tissue-engineered bone (PTEB) treated by recombinant human bone morphogenetic protein-2 applied in vivo (rhBMP-2; BMP) has shown additional advantages over simple application of rhBMP-2 in orthotopic site [1,3]. The possibility of immunological rejection and contamination that exists with ex vivo tissue-engineered constructs can be eliminated by “endocultivated” tissue-engineered constructs, with the extra advantage of enhanced access to the vascularization for the in vivo engineered implant.

It is crucial to select the appropriate time to transfer the PTEB for repairing bone defects. If the “prefabrication” time allowed in ectopic site is too short, the PTEB could be too soft to withstand the fixation of titanium implants or insertion of screws later, and may even lack sufficient vascularization to retain viability during or after the transfer. If the “prefabrication” time is too long, remodeling and resorption of PTEB may occur, thus decreasing the volume of regenerated bone [4,5]. However, while in vivo monitoring of the morphology, osteoinductivity, vascularization and volume of PTEB remains difficult, SPECT/CT is potentially a method to overcome this challenge. To our knowledge, few studies have reported on the localization and measurement of hard tissue volume, bone density, and ossification of PTEB and rhBMP-2 implant by non-invasive method [6]. Here, we evaluated the value of SPECT/CT in monitoring the osteoinductivity and ossification of PTEB and orthotopic rhBMP-2 implants in a primate model, and the optimal time to transfer the PTEB for mandibular reconstruction was investigated.

## Materials and Methods

### Animals and Ethics Statement

All experiments were approvd by the Peking University Institutional Review Board (IRB) Laboratory Animal Welfare Ethic Branch (Approval number: LA200801R). Animal cares including housing, feeding, and environmental enrichment were conducted in accordance with standard operating procedures (SOPs) at the Experimental Animal Center of PLA general Hospital, Beijing. A team consists of veterinarian and nurse managed the drugs and medications in the operation. The surgeries have been performed by a team of oral and maxillofacial surgeons. We carried out the animal experiments under the guidelines published in the Guide for the Care and Use of Laboratory Animals of the National Institutes of Health. Nine adult rhesus monkeys (male, 6–9 years old, and 6–12 kg) were used in the experiment. Briefly, the animals were individually housed in stainless steel cages under a 12 hours light-dark cycle at 25–27°C. The monkeys interacted with human everyday. Animals were fed a diet of vitamin enriched biscuits, fruits including banana, watermelon and etc, provided 1 L of water or drink water via behavioral tasks until they were satiated. Toys also were given to them. All animals were closely monitored on a regular basis throughout the day by several researchers as well as animal care staff in order to ensure that levels of health and welfare were maintained. The monkeys were housed and fed for 9 months following standard regulations. General anesthesia of rhesus monkey was performed as the following procedures. First, ketamine was injected intramuscularly or subcutaneously (20 mg/kg). Then, the general anesthesia was maintained by pentobarbital (1–2%, intravenously). The plus oxygen saturation was monitored by a monitor (PM-7000, Mindray® Patient Monitor, Shenzhen, China). Endotracheal intubation was performed by a trained veterinarian. Tramadol (50 mg) was given intramuscularly after surgical intervention to alleviate suffering. Animals were sacrificed at 26 weeks after implantation in a manner consistent with the recommendations of the American Veterinary Medical Association (AVMA) Guidelines on Euthanasia and Primate Center SOPs (IV administration of pentobarbital overdose). The size of the cages used in our study was 85 cm × 92 cm × 100 cm.

### Experimental Design

The rhBMP-2 was manufactured by the Genetic Institute of Huadong Medicine (Hangzhou, China). The demineralized freeze-dried bone allograft (DFDBA) and coralline hydroxyapatite (CHA) carriers for rhBMP-2 were manufactured by OsteoRad Biomaterial Co. (Taiyuan, China) and Yihuajian Commercial Co. (Beijing, China), respectively. The sustained delivery of rhBMP-2–incorporated DFDBA and CHA was described previously [3]. Briefly, 72 mg rhBMP-2 was dissolved in gelatin solution in acetic acid (30 mg/ml). One block of DFDBA or CHA was soaked in 1 ml gelatin solution with rhBMP-2 (1.5 mg/ml) and then freeze-dried in frozen tubes and kept at 2–7°C until use.

**Fig 1:**
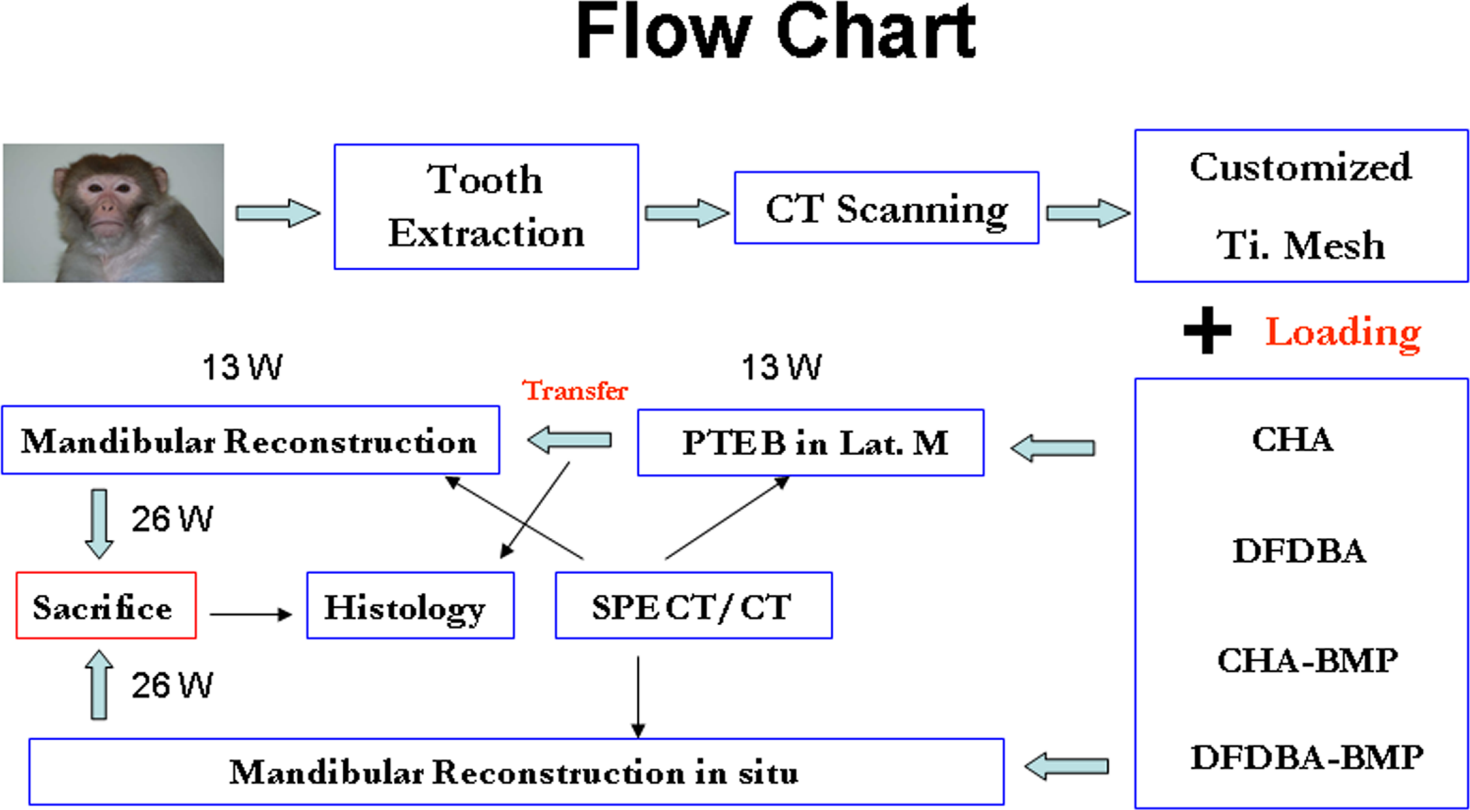
Flow chart of the experiment. In the prefabricated group, all the implants were placed in latissimus dorsi muscle for 13 weeks. Then, successfully endocultivated PTEB in group P-D-B and P-C-B were transferred to mandibular defect for another 13 weeks. Mandibular defects were reconstructed orthotopically by carriers with and without rhBMP-2 for 26 weeks.

Four kinds of constructs (DFDBA-BMP, CHA-BMP, DFDBA, and CHA) were evaluated for their capacity to support bone regeneration after implantation at ectopic or orthotopic sites. Four prefabricated groups [P-DFDBA-BMP (abbr. P-D-B), P-CHA-BMP (abbr. P-C-B), P-DFDBA (abbr. P-D), and P-CHA (abbr. P-C), n = 3 each] were applied according to the constructs applied. The constructs were loaded into customized titanium meshes and placed in the bilateral latissimus dorsi muscle of 6 monkeys. After 13 weeks, bone regeneration in P-D-B and P-C-B implants was observed; however, in the P-D and P-C groups that lacked BMP, no bone regeneration was found. Then, the successfully endocultivated PTEB flaps were transferred to repair mandibular defects. Six orthotopic groups [P-D-B, P-C-B, O-DFDBA-BMP (abbr. O-D-B), O-CHA-BMP (abbr. O-C-B), O-DFDBA (abbr. O-D), and O-CHA (abbr. O-C), n = 3 each] were evaluated in 18 mandibular defects. CHA and DFDBA with or without rhBMP-2 loading in customized meshes were placed in created mandibular defects. Clinical evaluation, SPECT/CT and histological examinations were performed to evaluate the osteoinductivity and bone regeneration of the PTEB and orthotopically placed implants. Twenty-six weeks after implantation, all monkeys were sacrificed by an overdose of sodium phenobarbital (Fig 1).

### Surgical Procedures

Surgical procedures were as described previously [3]. All animals followed the similar protocols: 2-cm long mandibular osteo-periosteal defects were created by mandibulectomy to serve as the orthotopic sites and latissimus dorsi muscle was prepared for prefabrication. Guided by computed tomography scanning of the animals’ heads, custom titanium meshes were fabricated by a milling machine (GSVM6540, Gold Sun Mould and CNC Machinery Co. Ltd., Guangzhou, China). Under general anesthesia, titanium meshes loaded with carriers with or without rhBMP-2 incorporated were implanted into the latissimus dorsi muscle of each monkey. In the meantime, mandibular defects were reconstructed with customized titanium mesh loaded with previous described constructs. One of 4 kinds of scaffold was loaded in each customized titanium mesh. Thirteen weeks later, PTEB were transferred for mandibular reconstruction with a pedicle containing the thoracodorsal artery and vein to repair the mandibular defects. During the transplantation, biopsy of PTEB was made. Implants without rhBMP-2 were incapable of osseoinduction ensuing ectopic implantation. As a result, P-D and P-C implants were not transferred to mandibular defects, and were instead harvested for histological examination only. For mandibular defects that were reconstructed with implants in situ, the titanium meshes loaded with P-D-B, P-C-B, O-D-B, O-C-B, O-D, or O-C constructs were fixed onto stumps of the mandibular defects with titanium micro-osteosynthesis screws (Fig 1).

### SPECT/CT Image Acquisition

The osteoinductivity and bone regeneration inside the meshes at 1, 4, 8, 13 and 26 weeks after implantation, and the viability of PTEB at 1 week after the transferring were evaluated using ^99m^Tc-MDP SPECT/CT. The SPECT/CT system (Symbia T6, Siemens, Germany) consisted of a dual-head variable-angle gamma (γ-) camera, equipped with low-energy, high-resolution collimators and a spiral CT (6 slices). Under general anesthesia, the monkey was positioned supine under the γ-camera on the imaging table after two hours latter of intravenous injection of 370 MBq of ^99m^Tc-methylene diphosphonate (^99m^Tc-MDP), tomographic imaging of the mandible and latissimus dorsi muscle was performed with a matrix size of 128 × 128, at 25 seconds per projection for 60 projections in steps with each image at a 6° angle.

After scintigraphic examination, CT (130 KV, 17 mAs) images were generated in 5 mm slices using an Esoft 2000 application package. With attenuation and scatter corrections, the integrated images were visualized in sagittal, coronal, and axial slices.

### SPECT/CT Image Interpretation

All the images were reviewed for orthotopic and ectopic ^99m^Tc-MDP concentration by two experienced nuclear medicine physicians independently at first. Then they reached the final image interpretation by consensus. The images were read in the following order: SPECT, CT and SPECT/CT. The density and volume of PTEB were calculated in the sagittal slices. The volume of interest over each titanium mesh in the latissimus dorsi muscle or mandibular defect was outlined, and the density and hard tissue volume of PTEB were calculated using CT. The uptake ratio of ^99m^Tc-MDP (T/PT) between the implant (T) and the vertebrae parallel to the implant in the same axial (for ectopic implants) or coronal slice (for orthotopic implants) (PT) was determined in a semi-quantitative analysis using a ROI (region of interest) technique by SPECT/CT [7].

### Histology

During the transplantation, biopsy of each implant in the latissimus dorsi muscle was obtained for histological analysis. Twenty-six weeks after implantation, mandibles including the titanium meshes were harvested and clinically examinated for its continuity. Then, the specimens were fixed in 10% neutral buffered formalin, and subsequently plasticized after dehydration using Technovit 7200VLC (Heraeus-Kulzer, Wehrheim, Germany). After solidification for 24 hours, the specimens were cut into slices along the long axis of the mandible approximately 40 to 80 μm in thickness and mounted on glass slides as previously described [3]. Two sections from each specimen block, 300 μm apart, were stained with hematoxylin and eosin (H&E). Bone regeneration of PTEB was calculated at 13 weeks after implantation. For mandibular specimen, the bone regenerated per defect area was calculated. The result of histology served to verify and test the reliability of findings from SPECT/CT.

### Statistical Analysis

Data from SPECT/CT were analyzed by one-way ANOVA by SPSS v15.0 (SPSS Inc., Chicago, IL). Differences among treatments were assessed by the Student-Newman-Keuls test. *P* < 0.05 was considered to be statistically significant.

## Results

### Animals and Clinical Investigation

All monkeys withstood the entire surgical procedure and the SPECT/CT examination. All wounds healed well and no infections developed at any of the surgical sites. Constant seroma was observed in local sites with rhBMP-2 applied, and it disappeared about 1 month after implantation. No necrosis of the transferred PTEB was found in any specimen.

### SPECT/CT and Histological Findings of the Prefabricated Groups

**Fig 2:**
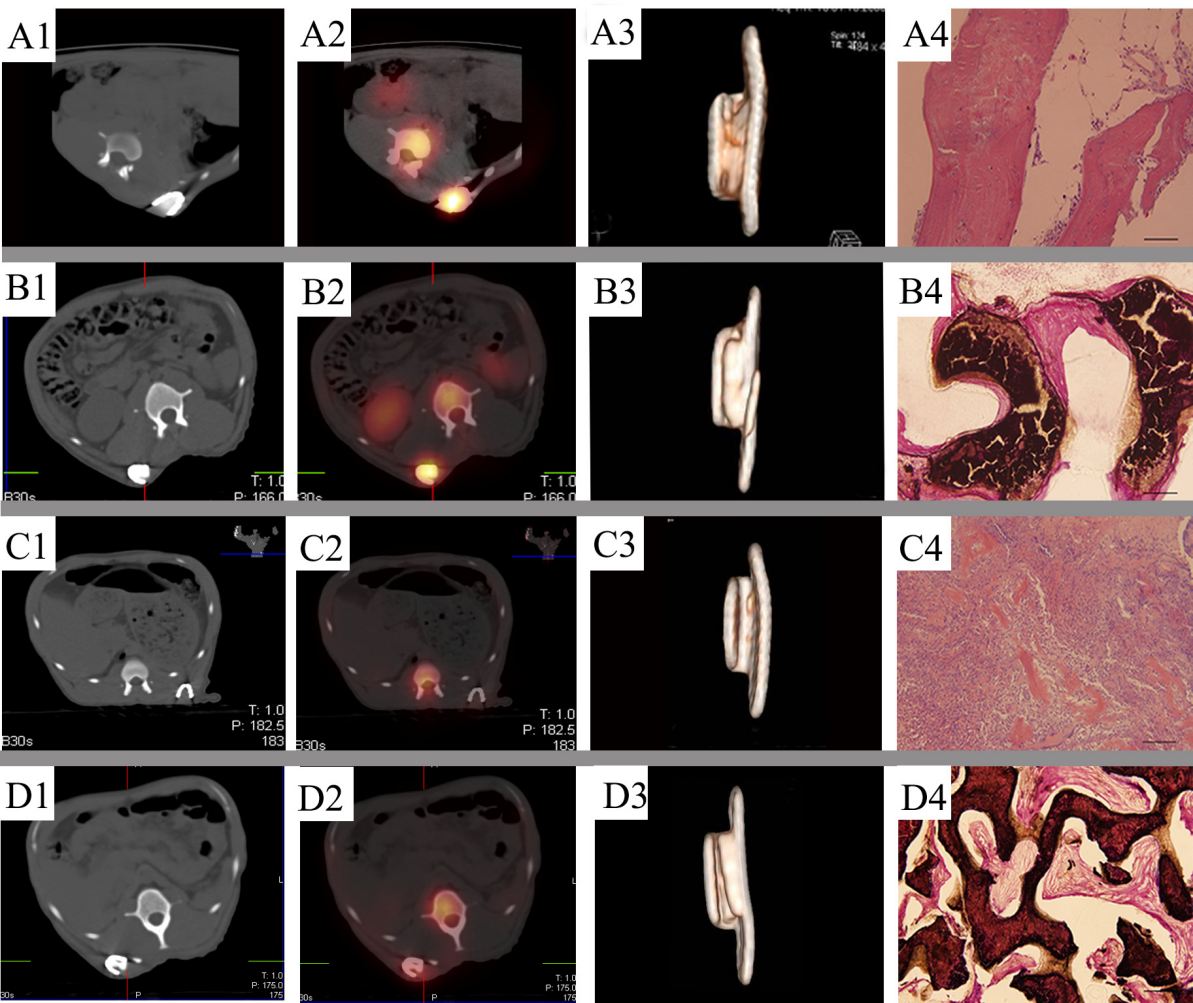
SPECT/CT, 3D CT and histology of ectopic implants in the latissimus dorsi muscle at 13 weeks after implantation. A_1_, B_1_, C_1_, and D_1_: Representative photographs of ectopic implants in CT images. A_2_, B_2_, C_2_, and D_2_: Representative photographs of ectopic implants in SPECT/CT fused images. A_3_, B_3_, C_3_, and D_3_: Representative photographs of ectopic implants in CT reconstructed images. A_4_, B_4_, C_4_, and D_4_: Histology of ectopic implants (H.E., Bar: 100 μm (A4, B4 and C4), 200μm (D4)).

**Fig 3:**
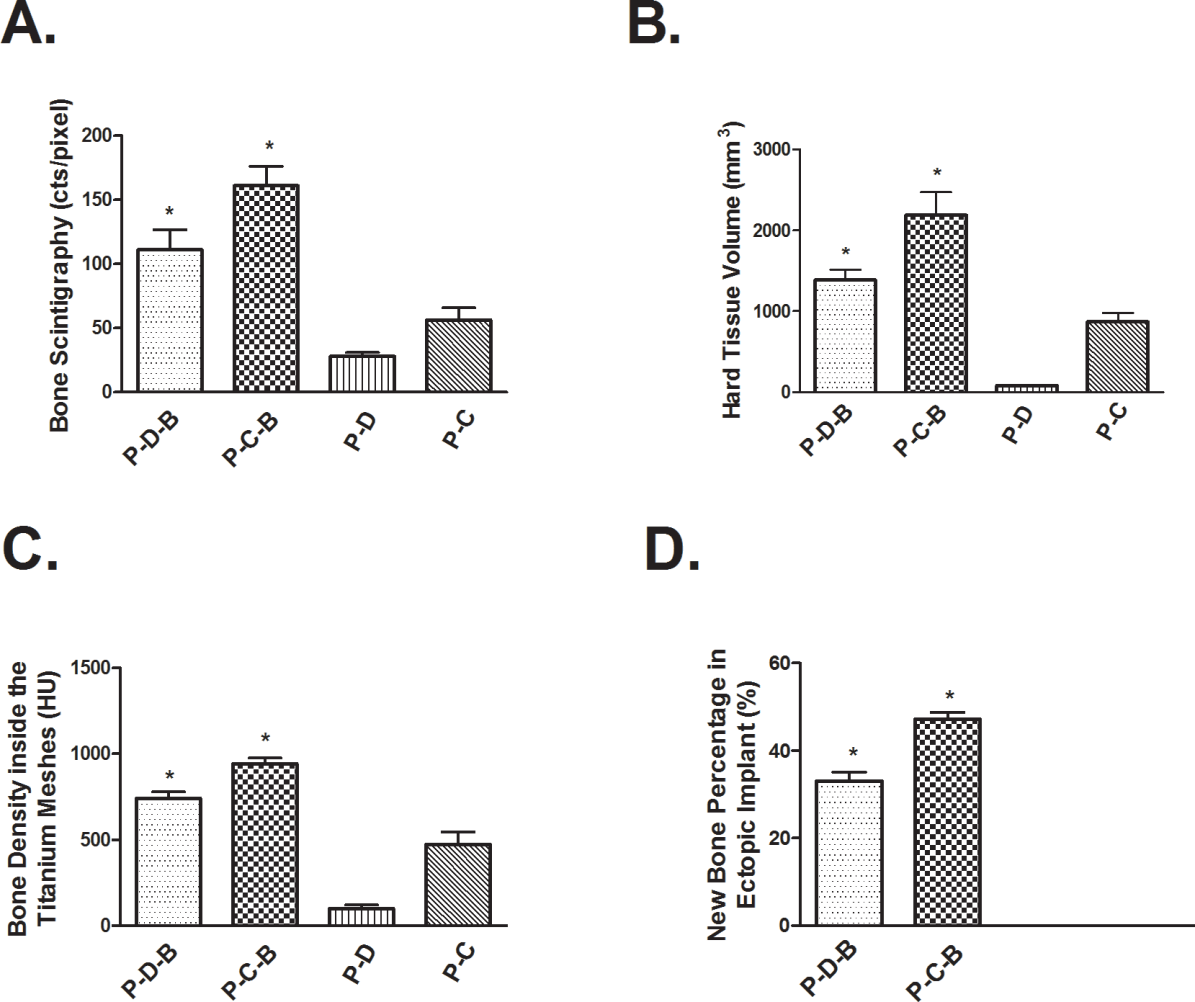
Results of SPECT/CT and histology of ectopic implants in the prefabricated groups at 13 weeks after implantation. A: Hard tissue volumes, as calculated from formatted CT images. B: Uptake ratio of the ectopic implants in titanium meshes, using ^99m^Tc-MDP SPECT/CT. C: Bone density of the ectopic implants in custom meshes, as calculated from formatted CT images. D: Quantification of bone regeneration of the ectopic implants in custom meshes by histological examination.

One week after implantation, the ectopic implants in prefabricated groups showed little ^99m^Tc-MDP uptake and there was no statistically significant difference among them (*P* > 0.05) (Table 1). Tracer uptake of the ectopic implants was much lower than that of the spine and mandible in the same axial slice, indicating that the uptake of ^99m^Tc-MDP was affected by the trauma of surgical intervention or the lower vascularization of the implants. Comparing to significant tracer uptake in the ectopic P-D-B (Fig 2A1 and 2A2) and P-C-B (Fig 2B1 and 2B2) implants, there was no significant tracer uptake in the ectopic P-D (Fig 2C1 and 2C2) and P-C (Fig 2D1 and 2D2) implants at 13 weeks after implantation (Fig 3A) (S1 Fig). The P-C-B implants showed statistically greater uptake of ^99m^Tc-MDP than the P-D-B implants from 4 to 13 weeks, indicating that the ectopic P-C-B implants had greater osteoinductivity (Table 1). At 8 weeks, the tracer uptake ratio of ^99m^Tc-MDP (T/PT) between the implants (T) and the vertebrae in same axial slice (PT) in P-D-B and P-C-B groups peaked, and then decreased. The T/PT values in these 2 groups were larger than 1.0 (Table 1). Reconstructed CT image showed bone formation in P-D-B (Fig 2A3) and P-C-B (Fig 2B3) implants and no bone regeneration in P-D (Fig 2C3) and P-C (Fig 2D3) from 4 to 13 weeks after ectopic implantation. The Hounsfield value of P-D-B implants, a measurement of bone density, increased gradually and reached the level of cancellous bone at 13 weeks after implantation (Table 2). The Hounsfield value of P-C-B implants decreased initially from 1 to 4 weeks, but rose to the level of cortical bone at 13 weeks (Fig 3C) (Table 2). There was a greater volume of ectopic bone regeneration in P-C-B (Fig 2B3) implant compared to P-D-B (Fig 2A3) and the other prefabricated groups at 13 weeks after implantation (Fig 3B) (Table 3), indicating that rhBMP-2 was active potent osteogenic inducer and the P-C-B implants proved to be the most efficient construct in the study. Consistently, histological examination (Fig 2A4, 2B4, 2C4 and 2D4) also confirmed significantly greater bone regeneration of ectopic P-C-B implant than that of P-D-B and other ectopic groups at 13 weeks after implantation from 3D reconstructed CT image (Fig 3D). For ectopic implants without rhBMP-2, the uptake ratio of ^99m^Tc-MDP remained low while the increase in the uptake ratio of ^99m^Tc-MDP of P-D implants was not statistically significant throughout the prefabrication period (*P* > 0.05) (Table 1). Furthermore, P-C implants showed little tracer uptake at 1 week after implantation. Tracer uptake increased in P-C implants from 1 to 4 weeks and stabilized from 8 to 13 weeks after implantation (Table 1). During the same period, density of the P-C implants in the custom meshes decreased (Table 2). Histology of biopsy from the P-C implants and P-D implants showed no bone regeneration (Fig 2C4 and 2D4).

**Table 1:** The uptake ratio of ^99m^Tc-MDP in the constructs of prefabricated and orthotopic groups (Mean ± SD).

Groups	1 w	4 w	8 w	13 w	14 w	26 w
P-D-B	0.17 ± 0.03	0.80 ± 0.10	1.38 ± 0.17	1.11 ± 0.15	4.24 ± 0.45	3.43 ± 0.31
P-C-B	0.16 ± 0.04	1.10 ± 0.13	1.98 ± 0.11	1.62 ± 0.15	4.78 ± 0.68	3.86 ± 0.29
P-D*	0.18 ± 0.03	0.29 ± 0.02	0.27 ± 0.03	0.28 ± 0.03	Not determined	Not determined
P-C*	0.18 ± 0.03	0.37 ± 0.07	0.58 ± 0.09	0.56 ± 0.09	Not determined	Not determined
O-D-B	1.51 ± 0.24	3.14 ± 0.78	3.95 ± 0.53	2.95 ± 0.25	Not determined	1.83 ± 0.24
O-C-B	1.78 ± 0.41	3.67 ± 0.59	4.26 ± 0.36	3.15 ± 0.38	Not determined	2.56 ± 0.31
O-D	0.37 ± 0.05	0.51 ± 0.16	0.69 ± 0.21	0.61 ± 0.23	Not determined	0.57 ± 0.18
O-C	0.35 ± 0.03	0.59 ± 0.10	0.74 ± 0.25	0.71 ± 0.25	Not determined	0.65 ± 0.14

*The prefabricated constructs of P-D and P-C groups were not transferred to mandibular defects.

**Table 2:** The density (Hounsfield units) in the constructs of prefabricated and orthotopic groups (Mean ± SD).

Groups	1 w	4 w	8 w	13 w	26 w
P-D-B	265.23 ± 31.25	461.57 ± 51.37	634.52 ± 72.38	740.39 ± 37.46	990.26 ± 67.29
P-C-B	574.76. ± 54.27	483.31 ± 53.35	788.92 ± 81.24	942.16 ± 33.88	1105.67 ± 54.18
P-D*	254.18 ± 27.48	211.45 ± 22.47	164.27 ± 18.21	100.89 ± 20.02	Not determined
P-C*	556.29 ± 51.36	434.87 ± 44.91	427.16 ± 45.73	312.01 ± 33.85	Not determined
O-D-B	271.81 ± 29.84	351.24 ± 39.82	436.64 ± 56.37	525.83 ± 57.48	602.12 ± 63.29
O-C-B	561.96 ± 51.37	635.82 ± 57.94	728.32 ± 74.82	839.92 ± 83.49	982.95 ± 68.46
O-D	261.80 ± 25.54	310.39 ± 43.21	351.32 ± 32.46	382.37 ± 41.23	418.25 ± 45.24
O-C	575.93 ± 59.79	553.28 ± 45.89	534.74 ± 45.62	523.83± 43.81	515.75 ± 49.09

*The prefabricated constructs of P-D and P-C groups were not transferred to mandibular defects.

**Table 3:** The hard tissue volume (mm3) in the constructs of prefabricated and orthotopic groups (Mean ± SD).

Groups	1 w	4 w	8 w	13 w	26 w
P-D-B	169.34 ± 17.46	363.57 ± 42.58	939.23 ± 68.91	1383.70 ± 90.16	2553.47 ± 203.56
P-C-B	1478.26 ± 136.82	1635.29 ± 151.47	1937.68 ± 163.01	2185.37 ± 289.26	2727.39 ± 237.84
P-D*	172.67 ± 16.31	155.08 ± 14.25	111.67 ± 10.42	79.31 ± 7.45	Not determined
P-C*	1426.38 ± 151.15	1207.59 ± 146.39	1006.58 ± 135.92	872.29 ± 108.63	Not determined
O-D-B	192.53 ± 23.17	435.38 ± 52.39	651.94 ± 71.31	892.36 ± 92.47	1383.54 ± 167.25
O-C-B	1537.82 ± 41.67	1745.36 ± 139.64	1993.56 ± 103.35	2142.38 ± 264.39	2347.68 ± 151.72
O-D	191.72 ± 26.46	342.58 ± 38.75	478.37 ± 53.38	526.93 ± 59.73	596.34 ± 46.92
O-C	1516.72 ± 31.27	1429.83 ± 126.94	1372.45 ± 153.28	1232.16 ± 135.56	1177.56 ± 59.83

*The prefabricated constructs of P-D and P-C groups were not transferred to mandibular defects.

### SPECT/CT and Histological Findings of the Orthotopic Groups

**Fig 4:**
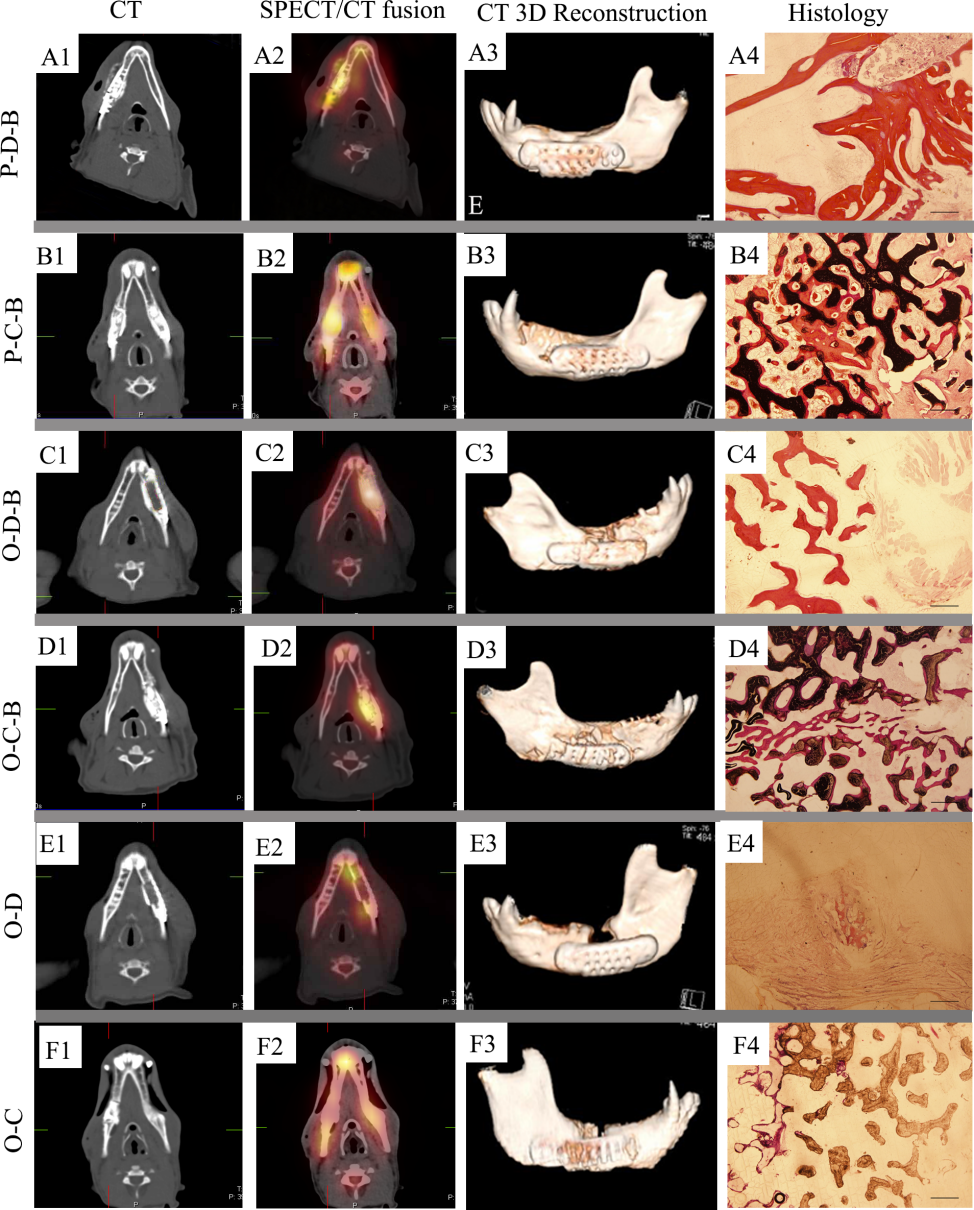
SPECT/CT of orthotopic implants in mandibular defects at 26 weeks after implantation, including two groups of transferred prefabricated tissue-engineered bone. A_1_, B_1_, C_1_, D_1_, E_1_, and F_1_: Representative photographs of orthotopic implants in CT images. A_2_, B_2_, C_2_, D_2_, E_2_, and F_2_: Representative photographs of orthotopic implants in SPECT/CT fused images. A_3_, B_3_, C_3_, D_3_, E_3_, and F_3_: Representative photographs of orthotopic implants in CT reconstructed images. A_4_, B_4_, C_4_, D_4_, E_4_, and F_4_: Histology of orthotopic implants (H.E., bar: 500 μm).

**Fig 5:**
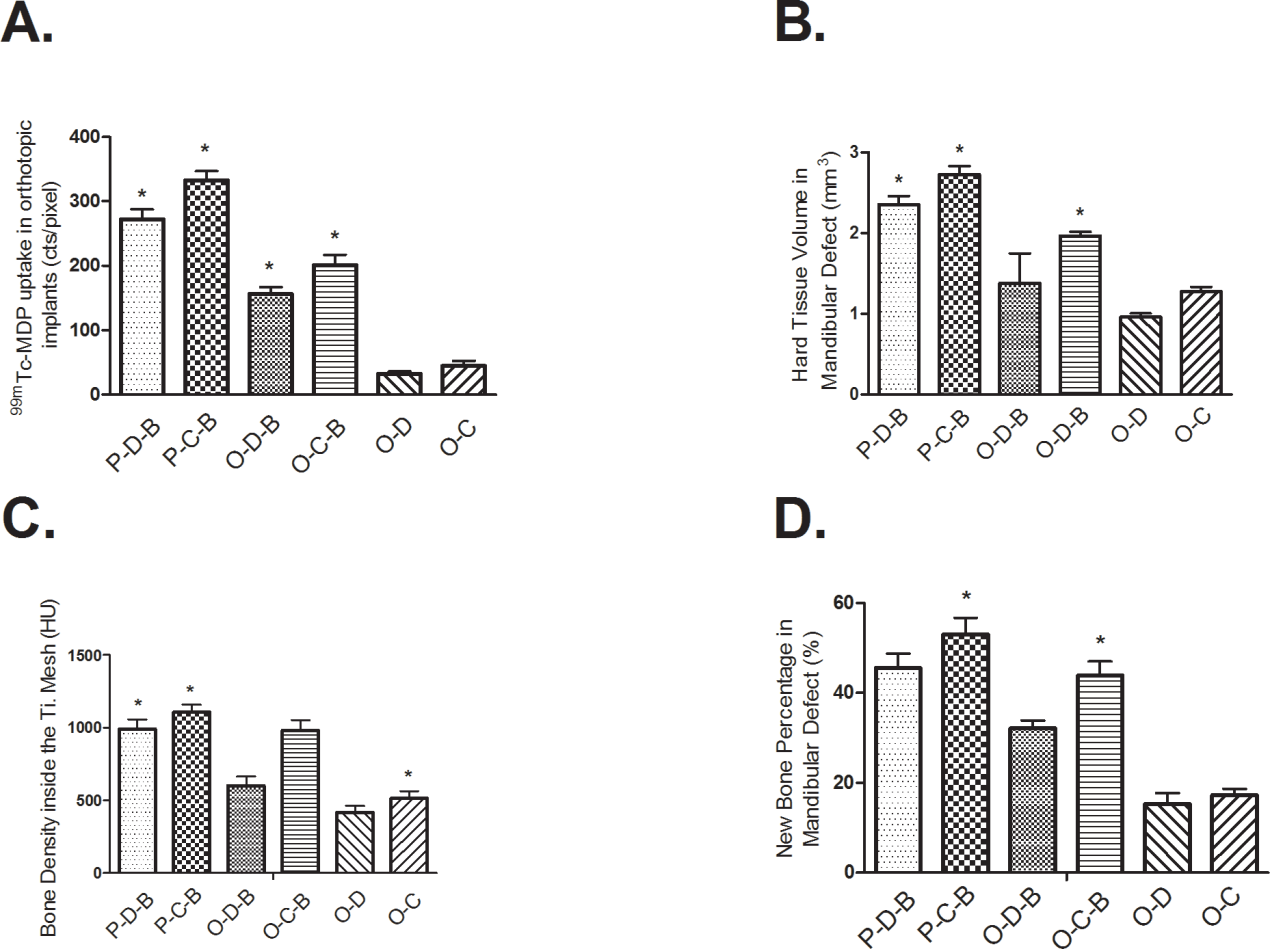
Results of SPECT/CT and histology of the implants in the orthotopic groups at 13 weeks after implantation. A: Hard tissue volumes, as calculated from formatted CT images. B: Uptake of the orthotopic implants in customized titanium meshes, using ^99m^Tc-MDP SPECT/CT. C: Bone density of the orthotopic implants in customized titanium meshes, as calculated from formatted CT images. D: Quantification of bone regeneration of the orthotopic implants in customized titanium meshes by histological examination.

One week after transferring, the P-D-B and P-C-B implants showed substantially increased ^99m^Tc-MDP uptake compared to the ectopic implants, indicating the viability and high osteoblasts activity of the transferred PTEB (Table 1). The ^99m^Tc-MDP uptake of P-D-B (Fig 4A1 and 4A2) and P-C-B (Fig 4B1 and 4B2) implants in mandibular defects was still higher than the other orthotopic groups (Fig 4C1, 4C2, 4D1, 4D2, 4E1, 4E2, 4F1 and 4F2) at 13 weeks after the transfer, suggesting continued regenerative ability. Particularly, the P-C-B implants showed the highest tracer uptake in the orthotopic groups (Fig 5A) (Table 1) (S2 Fig). Bone density of mandibular defect region in P-D-B, P-C-B and O-C-B groups increased significantly and was comparable to the host bone at 13 weeks after the transfer (Table 2). At that time, reconstructed CT image and histological examination showed the mandibular discontinuity was successfully repaired by P-D-B (Fig 4A3 and 4A4) and P-C-B (Fig 4B3 and 4B4) implants (Table 3). At 26 weeks after implantation, the bone volume and density of the transferred PTEB in mandibular defects were significantly greater than those from the orthotopic groups with rhBMP-2 applied in situ ((Fig 5B and 5C). The P-C-B (Fig 4B3) constructs showed the best bone regeneration in all the orthotopic groups (Fig 4A3, 4B3, 4C3, 4D3, 4E3 and 4F3) at 26 weeks after implantation (Fig 5D) (Tables 2 and 3).

^99m^Tc-MDP uptake could be detected in O-D-B and O-C-B implants from 1 to 26 weeks after implantation (Table 1). The ^99m^Tc-MDP uptake in O-D-B and O-C-B implants peaked at 8 weeks after implantation and then decreased (Table 1). The ^99m^Tc-MDP uptake was greater in the mandibular defects repaired with O-D-B and O-C-B implants compared to O-D and O-C implants (*P* < 0.05) (Table 1). O-C-B group showed greater ^99m^Tc-MDP uptake than O-D-B group from 4 to 26 weeks after implantation (Table 1). In O-D-B group, reconstructed CT image and histological examination revealed that the mandibular defect diminished, but the discontinuity of the mandible persisted after 26 weeks (Fig 4C3 and 4C4). The density, bone volume, and ^99m^Tc-MDP uptake of defected mandibles repaired with O-D-B were less than those with O-C-B or prefabricated bone (P-D-B and P-C-B) (Tables 1, 2 and 3). In O-C-B group, reconstructed CT image and histological examination showed newly formed bone osseointegrated with CHA blocks and the physical integrity of the mandible was restored at 26 weeks after implantation (Fig 4D3 and 4D4). Hard tissue volume at the mandibular sites of O-C-B implants were greater than that of O-D-B and the non-BMP orthotopic groups (*P* < 0.05), but it was less than those of the transferred, prefabricated constructs of the P-D-B and P-C-B (*P* < 0.05) (Table 3).

When O-D and O-C implants were placed in the mandible, little tracer uptake ensued (Table 1). Nearly half of the repaired mandible was not continuous in O-D group at 26 weeks after implantation. The density and hard tissue volume in mandibular defect in group O-D at 26 weeks was the lowest among all groups (*P* < 0.05) (Tables 2 and 3). Reconstructed CT image and histological examination confirmed the discontinuity of mandible in group O-D (Fig 4E3 and 4E4). O-C group showed higher ^99m^Tc-MDP uptake than O-D group from 4 to 26 weeks after implantation (Table 1). Reconstructed CT image revealed that bone regeneration was observed on the lingual side of the titanium mesh at 4 weeks after implantation. By 12 weeks, the mandibular defect had diminished, with notable bone regeneration on the lingual side and slight regeneration on the buccal side. However, at 26 weeks after implantation, the mandible defect was still noticeable with a gap remaining at the center, according to the CT image and histological examination (Fig 4F3 and 4F4). The density and hard tissue volume of mandibular defects in O-C group were larger than that in O-D group (*P* < 0.05) (Tables 2 and 3). Histological results confirmed that mandibular defects were repaired successfully with P-D-B, P-C-B implants or orthotopically with O-C-B implants, in accordance with SPECT/CT findings (Fig 5D)**.** Using prefabricated constructs worked better for bone reconstruction than application of rhBMP-2 in situ.

## Discussion

In the present study we have convincingly demonstrated that SPECT/CT is useful for evaluating the osteoinductivity of rhBMP-2 implant and monitoring the ossification of PTEB, thereby allowing accurate and case-by-case determination of the transfer timing for mandibular reconstruction.

SPECT is commonly used to monitor bone metabolism. However, it has limited resolution in delineating bone lesions as compared with CT which has more refined anatomical resolution [8]. As a result, we did not collect SPECT data of the implants accurately due to its undefined location. Hybrid SPECT/CT method combines both the functional and anatomical data in one image, allowing for better characterization and localization of skeletal lesions and for improved specificity in the imaging of bone lesions. It can also collect metabolic and morphological data from bone grafts during a single imaging session without needing to move the patient, thus reducing deviations in the registration of both datasets [9]. Also, SPECT/CT is more sensitive and less invasive than other methods (eg. X-ray, histology) in detecting the osteoinductivity of rhBMP-2 [6]. In our study, SPECT/CT effectively delineated the bone regeneration process, bone density, and tracer uptake of the PTEB without the need of invasive examination. When used to monitor bone regeneration by biomaterials implanted in vivo, SPECT/CT allows simultaneous evaluation of ectopic and orthotopic bone regeneration, and eliminates potential interference with bone induction and vascularization.

^99m^Tc-MDP SPECT/CT is also a valuable non-invasive tool in evaluating the bone regeneration process of orthotopic and ectopic implants comprising various types of carriers aided with rhBMP-2. We found no ^99m^Tc-MDP uptake and no bone regeneration from ectopic DFDBA implant in the muscle, suggesting that DFDBA has poor osteoinductivity in rhesus monkey. The reason may be that tissue environment becomes more specific in more evolved mammals and the osteoinductivity of DFDBA is decreased [10]. The ^99m^Tc-MDP uptake of CHA has been reported previously to take place via chemical absorption of nuclear tracer into the crystalline structure of hydroxyapatite [11]. The CHA-BMP implants yielded the highest uptake of the radioactive tracer and greatest bone regeneration among the prefabricated groups. It indicates that CHA could be taken as an ideal carrier for rhBMP-2. Contrary to the fast resorption of DFDBA, the slow resorption of CHA might enhance the osteoinductivity of rhBMP-2. The ratio of T/PT of rhBMP-2 incorporated carriers was larger than those carriers without rhBMP-2. We suggest that a ratio of T/PT larger than 1.0 as a criterion for proper tracer uptake of rhBMP-2 incorporated carriers; the ratio less than 0.7 as baseline for those carriers which attract ^99m^Tc-MDP by themselves, eg. CHA. The orthotopic rhBMP-2 implants yielded a higher ^99m^Tc-MDP uptake compared to the ectopic counterparts. To explain this difference, we suggest that the trauma of the mandibular resection and local tissue environment might activate the uptake of ^99m^Tc-MDP in situ.

The time of transferring such PTEB for bone reconstruction varied greatly in literature, depending on the variety of scaffolds, prefabrication sites, and rhBMPs [1,3,12]. Here, we propose transferring the PTEB containing rhBMP-2 at 8 to 13 weeks after implantation, which is basically consistent to studies from Warnke et al [1,13]. During this period, the bone density increased gradually and the uptake of ^99m^Tc-MDP used to monitor bone formation began to decrease from its peak at 8 weeks, implicating a slowdown of ossification after reaching maximal level. Our results showed that, with the ability to monitor several key parameters non-invasively, SPECT/CT provided a convenient yet accurate tool to determine and verify the optimal time point for transfer of ectopic prefabricated implants.

Due to limited number of ectopic and orthotopic implants, we could not harvest all the implants for histological examination at each time point for SPECT/CT examination. Since shorter prefabrication period is more economical and safer for clinical application, we did not extend monitored prefabrication beyond 13 weeks in this study.

The tumorigenicity of rhBMP-2 is a confusing issue for its application. The expression of BMP-2 is altered in solid tumors comparing to neighboring normal tissue, eg. increased expression in pleomorphic adenoma of salivary glands [14,15], non-small cell lung carcinomas [16] and reduced expression in microadenomas of familial adenomatous polyposis [17]. Recently, Wang et al found that BMP-2 plays a role to suppress tumor growth by reducing the gene expression of tumorigenic factors and inducing the differentiation of the stem cells in osteosarcoma and they suggested BMP-2 or BMP-2-mimetic drugs may therefore provide a new therapeutic option for treatment of osteosarcoma [18]. More investigations are needed to validate the correlation of BMPs and tumorigenicity. To be on the safe side, the PTEB is suggested to be used in the secondary bone defects in the patients with head and neck tumor who have observed for long time without recurrence.

## Conclusion

^99m^Tc-MDP SPECT/CT is a novel and non-invasive tool to monitor PTEB and orthotopically rhBMP-2 implants in live recipients. Mandibular reconstruction with PTEB achieved best bone regeneration, as compared to rhBMP-2 applied in situ. The PTEB achieved peak osteoinductivity and bone density at 8 to 13 weeks after implantation, which would serve as a suitable time frame for its transfer to mandibular reconstruction.

## Supporting Information

S1 Fig The representative photographs of the ectopic implants in prefabricated groups at 13 weeks after implantation in SPECT images. A: group P-D-B; B: group P-C-B; C: group P-D; D: group P-C (Arrows indicate the location of the ectopic implants).
(TIF)

S2 Fig The representative photographs of the orthotopic implants in mandibular defects at 26 weeks after implantation in SPECT images. A: group P-D-B; B: group P-C-B; C: group O-D-B; D: group O-C-B; E: group O-D; F: group O-C (Arrows indicate the location of the orthotopic implants).
(TIF)
