# Supplementary figures and images for: The Value of SPECT/CT in Monitoring Prefabricated Tissue-Engineered Bone and Orthotopic rhBMP-2 Implants for Mandibular Reconstruction
Source: PLoS One. 2015 Sep 4;10(9):e0137167. doi: 10.1371/journal.pone.0137167 (PMC4560383; doi:10.1371/journal.pone.0137167)

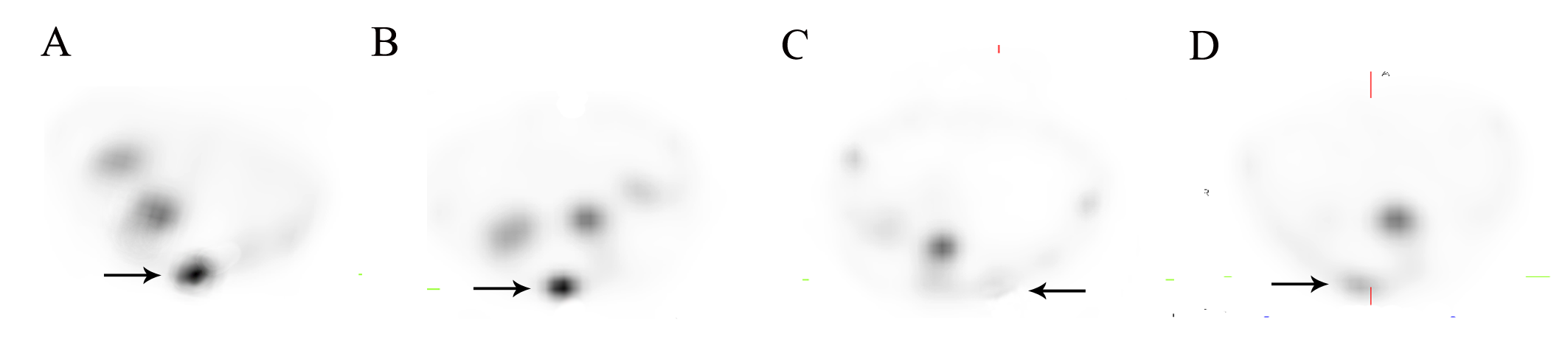

Supplement: S1 Fig — A: group P-D-B; B: group P-C-B; C: group P-D; D: group P-C (Arrows indicate the location of the ectopic implants). (TIF) [file pone.0137167.s001.tif]

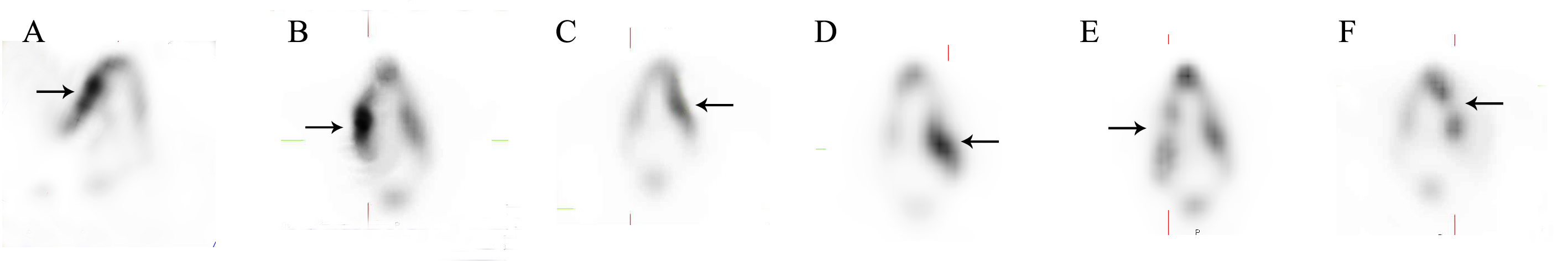

Supplement: S2 Fig — A: group P-D-B; B: group P-C-B; C: group O-D-B; D: group O-C-B; E: group O-D; F: group O-C (Arrows indicate the location of the orthotopic implants). (TIF) [file pone.0137167.s002.tif]
